# The joint use of ^99m^Tc-MAA-SPECT/CT and cone-beam CT optimizes radioembolization planning

**DOI:** 10.1186/s13550-021-00764-z

**Published:** 2021-03-04

**Authors:** Macarena Rodríguez-Fraile, Ana Ezponda, Fabiana Grisanti, Verónica Morán, Marta Calvo, Pablo Berián, Antonio Martínez de la Cuesta, Lidia Sancho, Mercedes Iñarrairaegui, Bruno Sangro, José Ignacio Bilbao

**Affiliations:** 1 Nuclear Medicine Department, Clínica Universidad de Navarra, Pamplona, Spain; 2 Radiology Department, Clínica Universidad de Navarra, Pamplona, Spain; 3 Medical Physics Department, Clínica Universidad de Navarra, Madrid, Spain; 4 Nuclear Medicine Department, Clínica Universidad de Navarra, Madrid, Spain; 5 Department of Internal Medicine-Hepatology, Clínica Universidad de Navarra, Pamplona, Spain

**Keywords:** Radioembolization (RE), CBCT, MAA, PET, Dosimetry, Target volume

## Abstract

**Purpose:**

To determine which imaging method used during radioembolization (RE) work-up: contrast-enhanced computed tomography (CECT), ^99m^Tc-MAA-SPECT/CT or cone beam-CT (CBCT), more accurately predicts the final target volume (TgV) as well as the influence that each modality has in the dosimetric calculation.

**Methods:**

TgVs from ^99m^Tc-MAA-SPECT/CT, CECT and CBCT were consecutively obtained in 24 patients treated with RE and compared with ^90^Y PET/CT TgV. Using the TgVs estimated by each imaging modality and a fictitious activity of 1 GBq, the corresponding absorbed doses by tumor and non-tumoral parenchyma were calculated for each patient. The absorbed doses for each modality were compared with the ones obtained using ^90^Y PET/CT TgV.

**Results:**

^99m^Tc-MAA-SPECT/CT predicted ^90^Y PET/CT TgV better than CBCT or CECT, even for selective or superselective administrations. Likewise, ^99m^Tc-MAA-SPECT/CT showed dosimetric values more similar to those obtained with ^90^Y PET/CT. Nevertheless, CBCT provided essential information for RE planning, such as ensuring the total coverage of the tumor and, in cases with more than one feeding artery, splitting the activity according to the volume of tumor perfused by each artery.

**Conclusion:**

The joint use of ^99m^Tc-MAA-SPECT/CT and CBCT optimizes dosimetric planning for RE procedures, enabling a more accurate personalized approach.

## Background

In radioembolization (RE), the definition of the target volume (TgV)—including tumoral and non-tumoral areas—that will receive the treatment, is decisive in many dosimetric aspects: for single-compartment medical internal radiation dose (MIRD) model because it assumes a uniform activity distribution within the TgV; for modified body surface area (mBSA) or partition model methods, because TgV is incorporated in the formulas [1]; for 3D voxel-dosimetry, because the dosimetric calculations derive precisely from the predicted TgV.

Current practice of assessing TgV is based on contrast-enhanced computed tomography (CECT) or magnetic resonance (MR), which reflect the standard anatomical venous segmentation as defined by Couinaud. However, this approach may be inaccurate in different clinical settings, such as in selective arterial (segmental and subsegmental) administrations, in *central* tumors without a pure lobar or segmental distribution, or in patients with anatomical variations—whether innate or related to tumorigenesis [2], among others.

Other imaging methods performed in the routine RE work-up have also been used to assess volumetric analysis. These include ^99m^Tc-macroaggregated albumin (^99m^Tc-MAA) SPECT/CT (^99m^Tc-MAA-SPECT/CT) or C-arm cone-beam CT (CBCT). The use of the ^99m^Tc-MAA-SPECT/CT as a method to calculate TgV was first described by Garin et al. [3], demonstrating its accuracy in hepatocellular carcinoma (HCC) [4] and in cholangiocarcinoma (CC)  [5]. Likewise, CBCT has been proposed as a useful method for defining the TgV in total or lobar administrations [6]–[8]. Rangraz et al. [7] demonstrated that using CBCT—instead of CECT—results in a difference in volumetric parameters However, none of the abovementioned studies has been performed in segmental or subsegmental administrations (treatment via direct tumor-feeding vessel) [9], where the evaluation of the TgV in the CBCT without clear anatomical limits may be more challenging.

Once the treatment is administered, both bremsstrahlung SPECT/CT (BS) or Yttrium-90 (^90^Y) PET/CT are generally used to verify the final distribution of the microspheres. Nevertheless, ^90^Y PET/CT has been shown to be superior to BS for the assessment of target activity [10], helping in the accurate quantification of the total delivered activity [11] and to perform dose estimation [12, 13]. Moreover, ^90^Y PET/CT-based dosimetry after RE with resin microspheres has been shown to predict outcome in patients with liver metastases from colorectal cancer (CC) [14]. Hence, ^90^Y PET/CT is a robust and reliable tool for the estimation of the 90Y-microspheres deposition.

The primary aim of this study was to determine which of the imaging methods available at the time of the initial evaluation of RE (CECT, ^99m^Tc-MAA-SPECT/CT or CBCT) predicts more accurately the TgV, having the ^90^Y PET/CT final TgV as the reference parameter. A secondary objective was to evaluate the influence that the differences in the estimated TgV for each technique has in the dosimetric calculation.

Finally, since in order to reduce the risk of RE-induced liver disease (REILD) [15], it is highly recommended to minimize the irradiation of the non-tumoral tissue [16, 17], RE administrations are becoming increasingly selective. In this sense, the contribution of ^99m^Tc-MAA-SPECT/CT and CBCT to the standard images (CECT) for a better dosimetric planning, especially in segmental or subsegmental approaches, was also analyzed [15].

## Materials and methods

### Same-day RE protocol

#### Patients

All patients treated with resin ^90^Y-microspheres (SIR-spheres®, SIRTex Medical Limited) in our center from October 2018 to April 2019 were consecutively studied.

After being considered as a candidate for RE by the hepatobiliary multidisciplinary team (MDT), a same-day planning and treatment was performed in all cases. Both the aim and the approach of the treatment (total, lobar, segmental or subsegmental) were always defined by the MDT.

#### Pre-treatment angiography

After the oral administration of 600 mg sodium perchlorate to block free ^99m^Tc-pertechnetate uptake by stomach [18], a 4F catheter was advanced via common femoral artery, and a selective angiography of both the superior mesenteric artery and the celiac trunk was performed. Coil embolization was performed, if necessary, to prevent the delivery of particles to the non-target tissue. The interventional radiologist (IR) performed in all cases the angiographic simulation to cover the entire tumoral tissue while preserving as much volume of non-tumoral parenchyma as possible. When multiple extra or intrahepatic vessels feeding the TgV were detected, a selective catheterization of each one was carried out. Thus, same-day flow redistribution was performed, when deemed necessary, to treat the complete tumoral area reducing the number of injection points [19, 20].

Diagnostic angiography and endovascular intervention were performed using the robotic digital subtraction angiography system (Artis Zeego Q, VE 40 A, Siemens Healthineers, Forchheim, Germany). CBCT was routinely performed immediately after the angiography to determine the best arterial access. It consisted of an unenhanced rotation (mask run) and contrast-enhanced rotations. Rotation time was 4 s. Parameters of CT acquisition were: tube voltage, 90 kV; 248 frames; 0.8º per frame; pixel size, 616 µm; acquisition time, 12 s. Once the selected arterial access was defined, 111–185 MBq ^99m^Tc-MAA was injected to mimic the future distribution of ^90^Y-microspheres.

TgV from CBCT image was delineated by a technician in radiology and supervised by the IR using a volume calculation software (Syngo DynaCT, Siemens Healthineers). The reconstruction used was as follows: voxel size 0.5 mm3 (full); slice matrix, 512 × 512; kernel type, HU (W 1400; C 550); 0.5 mm slice thickness; image characteristics, normal; reconstruction model, Nat Fill; viewing preset, Syngo Dyna CT. A 0.5 mm slice thickness was employed. Images were windowed to emphasize liver parenchyma (W 1400; C 550). Regions of interest (ROIs) were manually drawn every two axial images and then interpolated. The obtained volume was reviewed in sagittal and coronal dimensions and corrected if needed. For target volume delineation, MIP (maximum intensity projection) datasets were also employed. Edge enhancement was included in the volume determination. Tumor volume was more clearly visualized with MIP representation (6 mm).

The TgVs of CECT studies were obtained using Syngo.via software (Siemens Heathineers). CECT TgV was defined on cross-sectional images by a radiologists, using a fixed slice thickness (3 mm). The volumes of each slice were summed, independent of anatomical landmarks. Region of interest (ROI) were manually drawn in each slice involving the target/tumor volume. A unified window level (W, 300 HU) and window width (C, 40 HU) was determined. In all cases, tumoral volumes were also assessed by CECT images.

#### ^99m^Tc-MAA scintigraphy and SPECT/CT

Within 40 min after ^99m^Tc-MAA administration, planar scintigraphy and SPECT/CT (128 × 128, 180°, 64 projections, 20 s/projection) were performed (Symbia 2, Siemens Healthcare). The images were used to define: (a) the intrahepatic distribution of ^99m^Tc-MAA, (b) to calculate the hepatopulmonary shunt (HPS) and (c) to determine the tumor/non-tumor ratio (TNR), as described elsewhere [1]. A HPS, calculated on planar images, above 20% was considered a contraindication for the treatment.

**Fig. 1:**
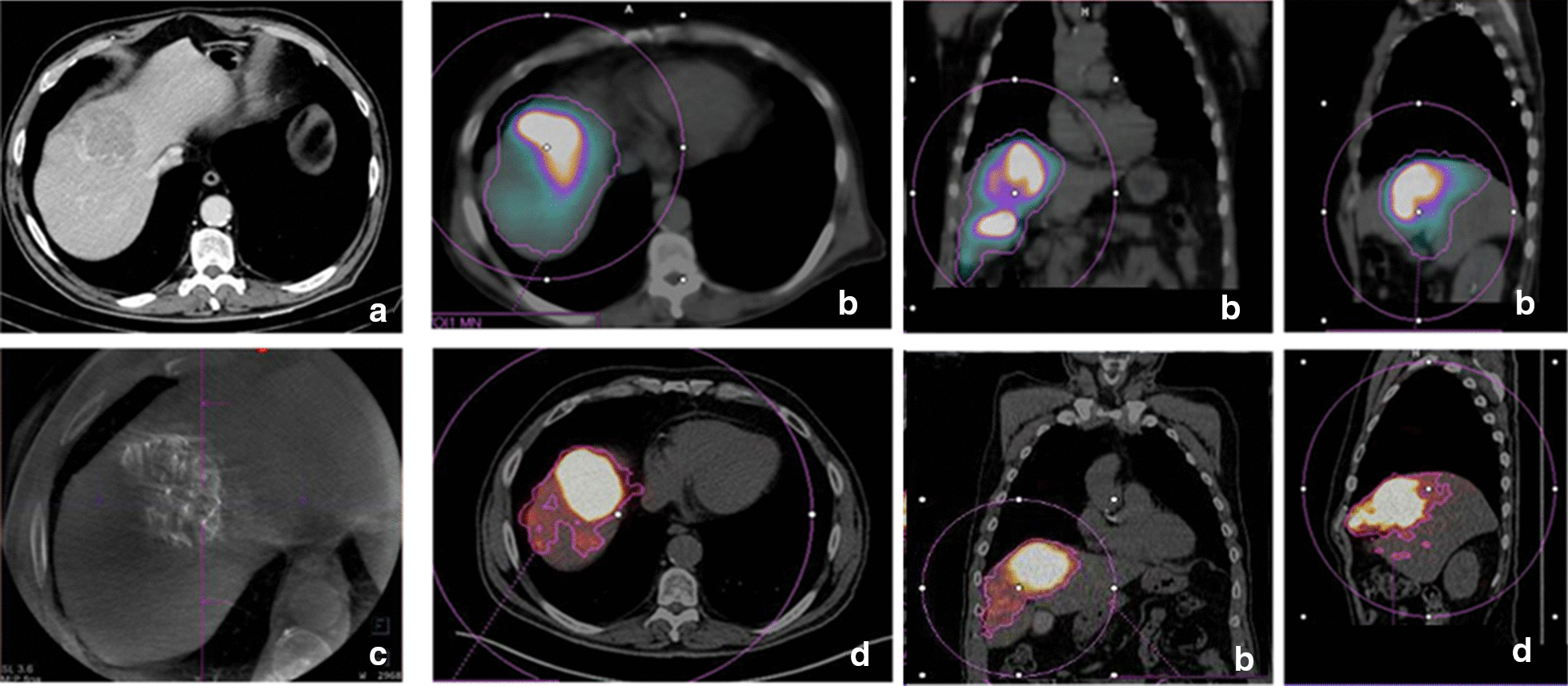
**a** Contrast-enhanced computed tomography (CECT) image in a patient with HCC located between segments IV and VIII. **b** Volumetric assessment of the target volume in SPECT/CT fusion images after the injection of ^99m^Tc-MAA through IV and VIII segments arteries. The volume was obtained using a “volume of interest and isocontour” tool, drawn in purple. **c** C-arm cone-beam CT (CBCT) showing contrast uptake in the tumoral lesion with no perfusion in non-tumoral parenchyma. **d** Volumetric assessment of the final target volume in the ^90^Y PET/CT fusion images

TgV in ^99m^Tc-MAA-SPECT/CT was defined using the multimodality reading software Syngo.via for MI (Siemens Healthineers). Using the “VOI + isocontour” tool, a volume of interest (VOI) in the target liver (including tumor and non-tumor) was drawn by a nuclear medicine (NM) physician and by means of the isocontour definition, the “molecular tumor volume or MTV” in milliliters (ml) obtained was used as the ^99m^Tc-MAA-SPECT/CT TgV. The isocontour threshold was visually adjusted to include the ^99m^Tc-MAA uptake volume into the VOI (Fig. 1).

#### Dosimetric calculation

The activities were calculated considering the absorbed doses (Gy) by tumor and healthy parenchyma as proposed by Gil-Alzugaray [21]. These absorbed doses were defined by formula [1] using the parameters obtained in the ^99m^Tc-MAA-SPECT/CT and CECT studies. In general terms, in cirrhotic patients with a predicted spared volume less than 40%, the activity was estimated to produce a safe absorbed dose by the non-tumoral compartment (≤ 40 Gy). In contrast, when the predicted TgV was small (< 60%) and the patient had a preserved liver function, a tumoricidal absorbed dose (> 100 Gy) was estimated, irrespective of the dose delivered to the non-tumoral tissue [21].

TNR values used for dosimetric calculations derived in all cases from ^99m^Tc-MAA-SPECT/CT.

#### Treatment administration

More than four hours after ^99m^Tc-MAA, resin ^90^Y-microspheres were administered during a new angiographic procedure. When ^99m^Tc-MAA-SPECT/CT showed an adequate distribution in the tumoral area, the vascular introducer was left in the same place for both procedures. In cases in which the arterial access selected by the IR did not optimally reach the tumoral area in the ^99m^Tc-MAA-SPECT/CT images, the angiography images were re-evaluated. In these cases, if a better access was identified, the site of injection between ^99m^Tc-MAA and RE differed. Although it is advisable to repeat ^99m^Tc-MAA evaluation when changes are made, it can be avoided if the modifications are minimal or do not have an impact on the safety of the treatment.

#### Post-treatment PET/CT

The morning after the RE treatment, a ^90^Y PET/CT scan centered on the liver region (two beds, 10 min/bed) was performed using a Biograph mCT-TrueV (Siemens Medical Solutions), which combines a 64-slice CT with a 21.8 cm field of view time-of-flight PET scanner comprised by lutetium-based crystals (LSO) detector blocks [22]. The reconstruction protocol used (one iteration, 21 subsets, a 6-mm Gaussian filter and a 200 × 200 matrix) was previously optimized by Martí-Climent et al. [11].

Final TgV in ^90^Y PET/CT was defined using the multimodality reading software Syngo.via for MI (Siemens Healthneers) as previously described for ^99m^Tc-MAA-SPECT/CT.

### Comparison of the TgV and the predicted dosimetry for each image method

TgVs calculated by ^99m^Tc-MAA-SPECT/CT, CECT and CBCT were compared with the TgV obtained in the ^90^Y PET/CT study.

In order to evaluate the influence that the differences that TgV for each technique has in the dosimetry, the absorbed doses calculated using the different TgV imaging modalities were compared using in all cases a fictitious prescribed activity of 1 GBq. The use of a fixed amount of activity makes it easier to appreciate the impact that the use of each TgV would have had on the absorbed doses. The TNR and tumor volumes were the real ones calculated for each patient. The absorbed doses for each modality were compared with the ones obtained using ^90^Y PET/CT TgV. Considering the latter as the actual ones [3, 4], the percentage of change between absorbed doses ([(Gy for ^90^Y PET/CT TgV – Gy for TgV modality)/Gy for TgV modality] × 100%), was calculated for each patient. A positive value indicates a percentage increase. (Gy in ^90^Y PET/CT is higher than the predicted using the TgV for each modality.) A negative value indicates a percentage decrease (Gy in ^90^Y PET/CT is lower than predicted using the TgV for each modality).

### Contribution of ^99m^Tc-MAA-SPECT/CT and CBCT for a better dosimetric RE planning

The contribution of CBCT and ^99m^Tc-MAA-SPECT/CT to the standard images (CECT) for a more personalized RE planning were evaluated by an IR and a NM physician (both with more than 15 years of experience in RE). The additional information provided by both techniques was especially focused on those clinical settings in which CECT may present some limitations for dosimetric calculations, such as selective or superselective approaches, several tumoral feeding arteries, flow redistribution, etc.

### Statistical analysis

To assess agreement between studies, the Lin Concordance Correlation Coefficient (CCC) and its 95% Confidence Interval (95% CI) were used. To define which study best predicts the final TgV, the determination coefficient (*R*^2^) from the regression model was utilized. For each modality, the difference changes of absorbed doses between treatment approaches were analyzed with the *t* students test. A *p* value less than 0.05 was used to determine the presence of a significant difference. The data were analyzed using Statistical Package for Social Science (SPSS) software version 22.

## Results

During the study period, 24 consecutive patients (18 men, 63.54 years (± 6.6)) underwent same-day RE with resin ^90^Y microspheres (20 HCC, three CC and one neuroendocrine tumor).

Flow redistribution was performed in 10 patients (41.7%), being the embolized vessels: phrenic arteries (*n* = 5), segment IV artery (*n* = 2), left gastric artery (*n* = 4), renal capsular artery (*n* = 1), gastroduodenal artery (*n* = 1) and middle hepatic artery (*n* = 1).

For cases with two or more supply arteries, ^99m^Tc-MAA activity was divided into 25%, 50% or 75% at the IR discretion, depending on the findings obtained during mapping arteriography. CBCT was performed in 23/24 patients (in 1/24 was not possible due to lack of patient collaboration). Four CBCT studies were excluded from the TgV analysis. An insufficient CBCT technique did not allow the correct assessment of TgV.

In all cases CBCT volumetry was obtained after ^99m^Tc-MAA injection, so the calculation was not used to split ^99m^Tc-MAA activity.

CECT and ^99m^Tc-MAA-SPECT/CT TgVs were calculated in all patients during RE planning, not knowing the final distribution of ^90^Y-microspheres on ^90^Y PET/CT (Table 1).

**Table 1:** ^99m^Tc-MAA and ^90^Y-microspheres injection sites, percentage of ^99m^Tc-MAA activity administered through every artery (decided by IR based on liver and tumor volumes) and split of ^90^Y-microspheres prescribed by each artery (in up to 42% of patients according to CBCT volumetric information)

Patient	^99m^Tc-MAA injection (%)	CECT TgV (ml)	CBCT TgV (ml)	MAA-SPECT/CT TgV (ml)	90Y-microspheres injection (prescribed) (GBq)	^90^Y-PET/CT TgV (ml)
1•	I (75), LHA (25)	640	626	640	I (0.75), LHA (0.25)	1445
2	VI (50), VIII(50)	392	NA	412	VI (0.5), VIII (0.2)	654
3•#	IV (50), VIII (50)	270	206	537	LHA (0.8), RHA (0.7)	1561
4•	IV (50) and VIII (50) subsegmental arteries	25	25	83	IV (0.3) and VIII (0.6) subsegmental arteries	405
5	IV	99	144	218	IV (1)	257
6	V–VIII (50), IV (50)	267	292	858	V–VIII (0.5), IV (1)	755
7	IV	135	70	133	IV (1)	181
8	VI–VII	276	228	451	VI–VII (1.5)	420
9	VIII (50), VI (50)	746	511	1127	VIII (1.1), VI (0.5)	1277
10	V-VIII (50), Inferior Phrenic artery (50)	340	480	707	V–VIII (0.65), Inferior Phrenic artery (0.65)	870
11	RHA (50), Right Inferior Phrenic artery (50)	1147	1515	1097	RHA (0.9), Right Inferior Phrenic artery (0.7)	1298
12•	IV	361	NA	350	Branches 1 (0.4) and 2 (0.4) of IV artery	399
13	VIII (33), VII (33) II (33)	960	NA	917	VIII (0.5), VII (0.5), II (0.2)	1118
14	V–VIII (75), IV (25)	537	NA	489	V–VIII (0.3), IV (0.7)	622
15	RHA (75), IV (25)	1550	1860	1276	RHA (1.2), IV (0.3)	1373
16	VIII (50), VII (50)	510	903	1260	VIII (0.7), VII (0.7)	1357
17	IV (75), II (25)	290	450	254	IV (0.65), II (0.25)	350
18	Accesory HA (42), Proper HA (35), IV (22)	1186	NA	1313	Accesory HA (1.7), Proper HA (1.3), IV (0.4)	1380
19#	RHA (25), LHA (75)	1955	1975	1468	RHA (0.4), LHA (1)	1536
20#	RHA	2260	2305	2279	RHA (3.4)	2420
21#	RHA (75), LHA (25)	1285	1387	1516	RHA (1), LHA (0.18)	1683
22#	RHA	760	974	986	RHA (0.82)	992
23#	RHA	700	971	802	RHA (0.64)	922
24#	RHA	580	966	795	RHA (1)	792

Target volumes obtained for each patient with CECT, CBCT, ^99m^Tc-MAA SPECT/CT as well as final target volume ^90^Y-PET/CT, are also reported

•Patients with intended or unintended changes between MAA and 90Y-microspheres administrations

^#^Pure lobar and total treatments

*RHA* Right Hepatic Artery, *LHA* Left Hepatic Artery, *NA* non available

Mean HPS was 6.9% (± 3.4). Mean TNR was 2.6 (± 1.5). In the majority of cases TNR was an average of the uptake in all tumors, while in 4 cases it was calculated for each tumor as described elsewhere [23]. To simplify the results, a mean of the TNR of all tumors was calculated in these 4 patients.

In 17/24 patients (71%), RE injection were performed through segmental or subsegmental arteries. Seven patients were treated with just one infusion of ^90^Y-microspheres, four were lobar (right) and three were segmental. Fifteen patients were treated from two different arteries, three of them received a whole-liver treatment (right and left hepatic arteries), two were treated through a lobar and a segmental branch, eight through two different segmental/subsegmental branches and two through the inferior phrenic artery in association with a lobar (1) or a segmental (1) artery. Finally, two patients required three different infusions through segmental and accessory arteries (Table 1).

In four patients (16%), as depicted in Table 1, ^99m^Tc-MAA-SPECT/CT and RE administrations differed due to patient motion during RE (Patient #4), vasospasm (Patient #1) or minimal deliberate changes to improve tumor coverage (Patients #3 and #12).

Median (interquartile range or IQR) administered activity was 1.2 (0.5–3.4) GBq, obtained in 23 patients by partition model formula and in one patient by BSA (− 20%).

### Comparison of the TgV and the predicted dosimetry for each image method

Medians TgVs were 558.5 (25–2260) ml for CECT, 626 (25–2305) ml for CBCT, 798.5 (83–2279) ml for ^99m^Tc-MAA-SPECT/CT, and 957 (181–2420) ml for ^90^Y PET/CT (Table 1).

Isocontour mode was 3% (range = 1–9%) for both ^99m^Tc-MAA-SPECT/CT and ^90^Y PET/CT. This value was used for the definition of TgV in 16/24 patients (67%) for ^99m^Tc-MAA-SPECT/CT and in 17/24 patients (71%) for ^90^Y PET/CT.

**Fig. 2:**
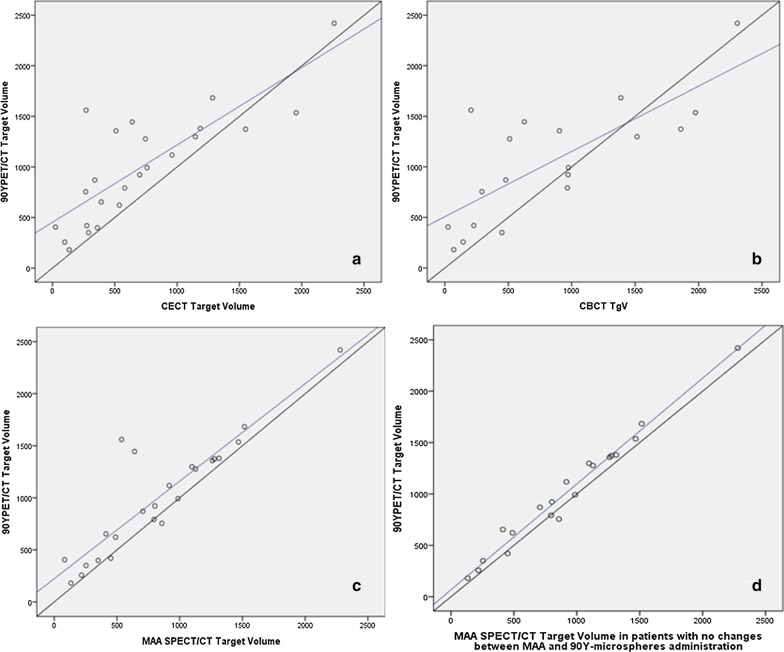
Correlation (blue line) between the target volume obtained with ^90^Y PET/CT and CECT (**a**), CBCT (**b**) and ^99m^Tc-MAA SPECT/CT in all patients (**c**) and excluding patients with changes between MAA and ^90^Y-microspheres administrations (**d**). Diagonal black line indicates perfect correlation between both variables

The concordance with ^90^Y PET/CT final TgV was substantial for CBCT (CCC = 0.71; 95% CI = 0.42–0.87) and for CECT (CCC = 0.72; 95% CI = 0.49–0.85). Maximal concordance was reached by ^99m^Tc-MAA-SPECT/CT (CCC = 0.85; 95% CI = 0.7–0.87). This concordance was even higher when those four patients with changes between ^99m^Tc-MAA and RE administration were excluded (CCC = 0.97; 95% CI = 0.94–0.99) (Fig. 2).

**Fig. 3:**
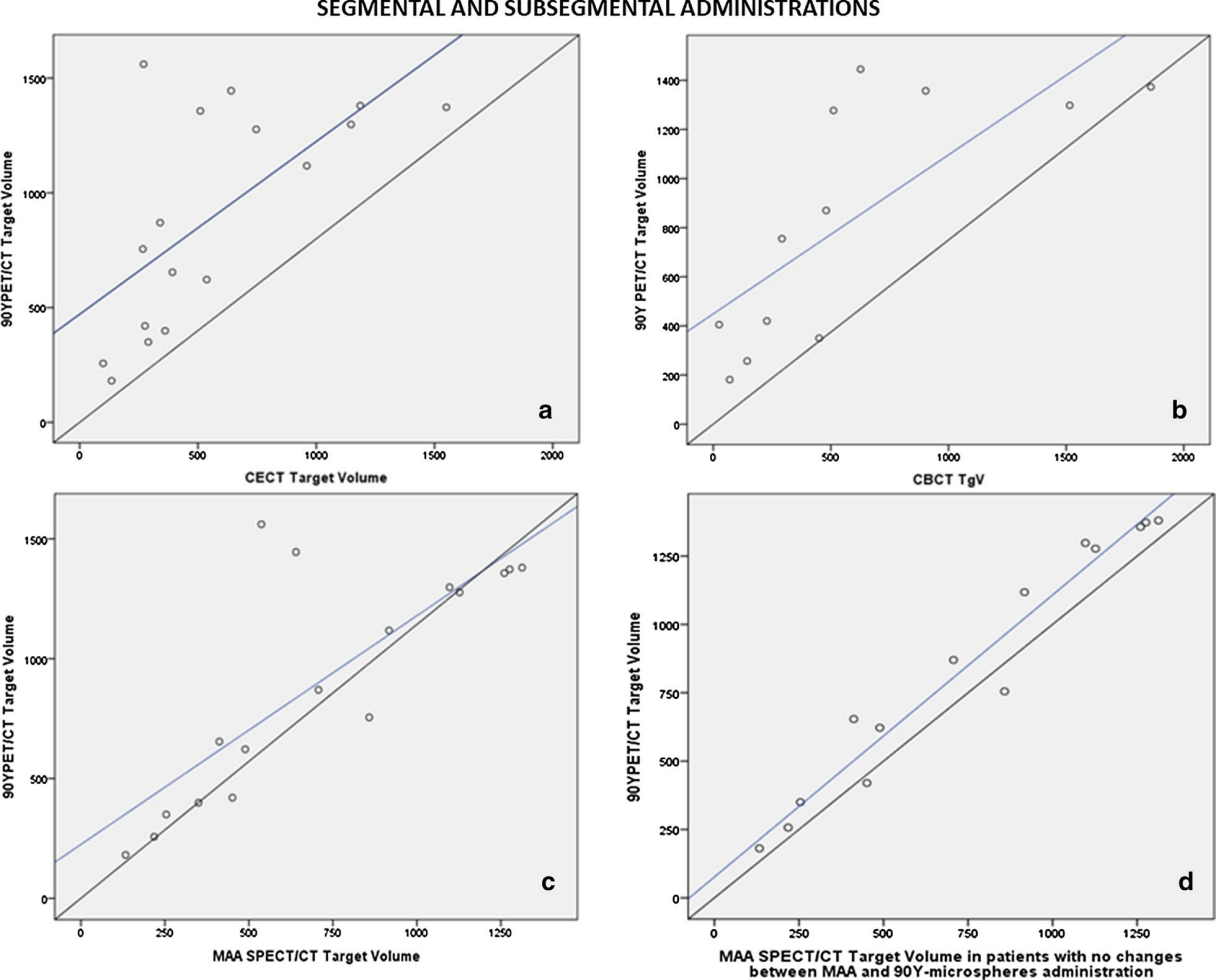
Correlation (blue lines) between the target volume obtained with ^90^Y PET/CT and CECT (**a**), CBCT (**b**) and ^99m^Tc-MAA SPECT/CT (**c**) in patients with segmental and subsegmental administrations. When patients with changes between ^99m^Tc-MAA and ^90^Y-microspheres administrations were excluded, ^99m^Tc-MAA correlation slightly improved (**d**). Diagonal black line indicates perfect correlation between both variables

When only segmental or subsegmental administrations were evaluated (*n* = 17), the concordance with ^90^Y PET/CT final TgV was moderate for CECT (CCC = 0.5; 95% CI = 0.2–0.74) while was substantial for CBCT (CCC = 0.67; 95% CI = 0.26–0.87) and for ^99m^Tc-MAA-SPECT/CT (CCC = 0.71; 95% CI = 0.42–0.87). Exclusion of the four patients with changes between ^99m^Tc-MAA and RE administration supposed a substantial improvement for ^99m^Tc-MAA-SPECT/CT (CCC = 0.95; 95% CI = 0.87–0.98) (Fig. 3) but minimal for CBCT (CCC = 0.73; 0.3–0.91).

The three studies predicted linearly the ^90^Y PET/CT final TgV. However, both CBCT (*R*^2^ = 0.66, *p* < 0.05) and CECT (*R*^2^ = 0.68, *p* < 0.01) showed a moderate weak *R*^2^, being strong for ^99m^Tc-MAA-SPECT/CT (*R*^2^ = 0.96, *p* < 0.01).

The median of the absorbed doses by tumor calculated using a fictitious activity of 1 GBq was 152 (98–250) Gy for CECT TgV, 125 (73–253) Gy for CBCT TgV, 116 (70–244) Gy for ^99m^Tc-MAA-SPECT/CT TgV and 102 (64–162) Gy ^90^Y PET/CT TgV. The median of the absorbed doses by non-tumoral liver using CECT was 74 (36–113) Gy, CBCT was 51 (31–104) Gy, ^99m^Tc-MAA-SPECT/CT was 46 (35–95) Gy and ^90^Y PET/CT TgV was 40 (30–75) Gy. The absorbed doses by tumor calculated using ^90^Y PET/CT showed a median difference with the ones predicted by CECT of − 33 (− 65 to − 13) Gy, by CBCT of − 20 (− 194 to 14) Gy and by ^99m^Tc-MAA-SPECT/CT of − 14 (− 54 to − 1) Gy. For non-tumoral liver of − 15 (− 38 to − 4) Gy using CECT, of − 17 (− 52 to 5) Gy using CBCT and of − 5 (− 37 to − 1) Gy using ^99m^Tc-MAA-SPECT/CT. These values represent a mean percentage of change between the absorbed doses obtained using ^90^Y PET/CT TgV and the ones predicted with CECT of − 29 (± 30)%, with CBCT of − 23 (± 38)% and with ^99m^Tc-MAA-SPECT/CT of − 18 (± 24)%. There were no statistically significant differences between the treatment approach (lobar and lobar extended/ total vs. selective and superselective) and the percentage of change for CECT and for ^99m^Tc-MAA-SPECT/CT. However, CBCT showed values of absorbed doses more similar to ^90^Y PET/CT for lobar and total approaches (− 2.5 ± 31%) than for selective (− 38 ± 37%) administrations (*p* < 0.05).

### Contribution of ^99m^Tc-MAA-SPECT/CT and CBCT for a better dosimetric RE planning

The information provided by ^99m^Tc-MAA-SPECT/CT was determinant in 17 patients (71%), due to its capability: (a) to predict 90Y-microspheres distribution in patients with segmental or subsegmental treatments (*n* = 8); (b) to confirm the TgV after flow redistribution (*n* = 7) and (c) to detect tumoral areas not covered with the selected arterial access (*n* = 2).

**Fig. 4:**
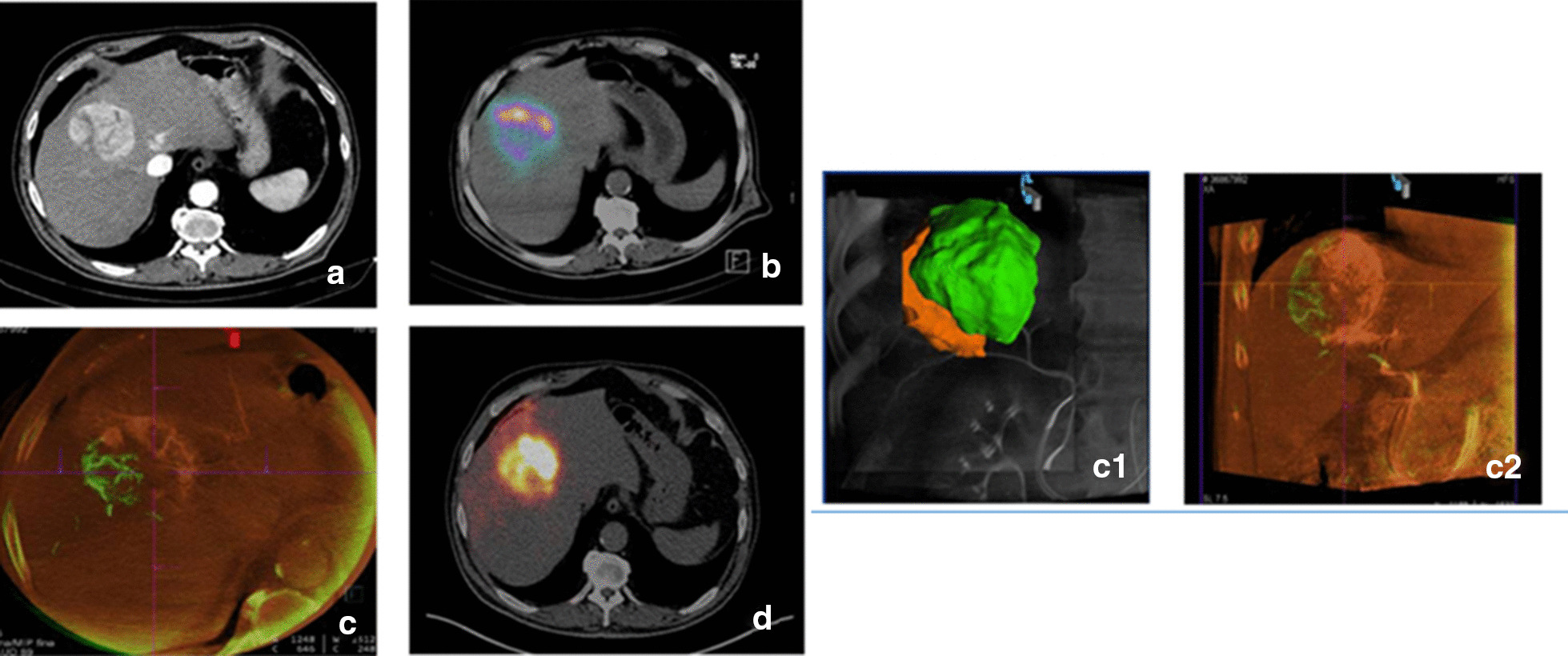
Same patient as Fig. 1. **a** Contrast-enhanced computed tomography (CECT) image: HCC located between segments IV and VIII. **b** ^99m^Tc-MAA SPECT/CT fusion image shows low uptake in the lateral part of the tumoral nodule. ^99m^Tc-MAA activity was split in two doses of 50% each by IR decision, based on liver and tumoral volumes. **c, c1** and **c2** C-arm cone-Beam CT (CBCT) volumetric assessment of the tumoral territory perfused by each artery. VIII segments artery (in green) fed only 32% of the tumoral volume while IV segment artery fed most of it (in orange). **d** ^90^Y PET/CT fusion image after splitting the activity according to CBCT volumes shows the adequate distribution of the microspheres throughout the lesion

*CBCT* helped to define the percentage of tumor volume perfused by each artery in 10 patients (42%) in whom the tumor was fed by more than one artery. This volumetric information was used to split the activity of ^90^Y-microspheres accordingly (Fig. 4). Moreover, CBCT allowed to ensure the tumor coverage in six patients (25%) and to rule out the presence of microsatellite lesions in one patient (4%). Globally, CBCT information was used for a personalized and more accurate planning in 17/24 patients (71%).

## Discussion

The results of this study show that ^99m^Tc-MAA-SPECT/CT predicts more accurately the final TgV—as defined in the ^90^Y PET/CT—than CBCT or even than the conventional method (CECT). This superiority is even more notable in segmental and subsegmental administrations. The differences obtained in the TgV for each method would have had a significant impact on the dosimetric calculation. Additionally, ^99m^Tc-MAA-SPECT/CT and CBCT information were determinant for RE planning in a significant proportion of patients. Our study therefore suggests that the joint use of both techniques optimizes dosimetry planning for RE procedures.

In this study, TgV obtained with different imaging modalities using the ^90^Y PET/CT as the method of validation, which has proven to be the most accurate technique for determining the final distribution of the microspheres [11, 12].

Since all consecutive patients treated with RE in our center in a period of time were studied, not only the optimal situations (identical ^99m^Tc-MAA and RE administrations) were included. As it occurs in daily practice, patients with intended or unintended modifications between both procedures were considered. Furthermore, flow redistribution was performed in 41.7% of the patients. Despite all these complex circumstances, the ^99m^Tc-MAA-SPECT/CT TgV reached maximal concordance with ^90^Y PET/CT final TgV (CCC = 0.85). As expected, when the four patients with changes between ^99m^Tc-MAA and RE administrations were excluded, ^99m^Tc-MAA-SPECT/CT increased its concordance value (CCC = 0.97). The high concordance found between ^99m^Tc-MAA-SPECT/CT and ^90^Y PET/CT volumetry supports the use of ^99m^Tc-MAA-SPECT/CT as the most reliable available tool for predicting final TgV. Although 3D voxel-dosimetry is currently recommended, the methodology followed here sustains its reliability even using a simple tool available by most groups performing RE.

In 71% of patients, RE administrations were performed through segmental or subsegmental arteries. When only these selective administrations were analyzed, ^99m^Tc-MAA-SPECT/CT showed to be superior to CECT (CCC = 0.5) and to CBCT (CCC = 0.67) for predicting TgV, with a substantial concordance (CCC = 0.71) with ^90^Y PET/CT TgV. These findings demonstrate that ^99m^Tc-MAA-SPECT/CT is also an effective tool for defining the TgV in segmental or subsegmental administrations, where CECT has some limitations [2, 8]. Because of the benefit to patient outcome of the parenchyma-sparing RE administrations [16, 17], selective administrations are recommended when possible. Hitherto, CECT volumes have been traditionally used for these selective administrations. However, and according to the results obtained in this study, CECT volumes poorly predict the final TgV obtained with ^90^Y PET/CT.

As for the reproducibility of ^99m^Tc-MAA-SPECT/CT and ^90^Y PET/CT isocontour definition, our results are comparable to previous studies: the isocontour mode was 3% (range 1–9%) for them both. This is in accordance with Richetta et al. [24] that using a mean threshold of 3% (range of 2–4%) found a good dose agreement between ^99m^Tc-MAA-SPECT/CT and ^90^Y PET/CT. Martí-Climent et al. [11] in a series of 10 patients found that 5% was the isocontour level that provided the lowest relative difference between reconstructed activity and activity delivered to the whole-liver ((10.2 ± 14.7)%). Moreover, Garin et al. [3], in a phantom study using an adaptative thresholding method based on SPECT/CT images, as used in our study, encountered a mean error of < 2.5% for volumes larger than 16 ml. Therefore, it seems that the visual adjustment of the isocontour level for the definition of the total treated liver is feasible and there are not significant variations on the value used between groups. It should be noted that all TgV ^99m^Tc-MAA-SPECT/CT were prospectively defined, not knowing the final microsphere distribution.

CBCT showed only a moderate concordance with ^90^Y PET/CT final TgV, lower than for ^99m^Tc-MAA-SPECT/CT. Although both are functional modalities, CBCT depends on the lapse of time between injection of the contrast agent and the image acquisition and also on the speed and volume of the injection. In some of our cases, this contrast volume could be insufficient to precisely demarcate the limits of the TgV. This was sometimes done deliberately to avoid contrast reflux to non-target areas or because CBCT was performed to detect other tumoral nodules—and not with volumetric purposes. Therefore, more studies are needed to discern whether CBCT moderate accuracy encountered was due to a limited capacity to define TgV in segmental/subsegmental administrations without clear anatomical limits or due to technical issues. Nevertheless, CBCT has other advantages not explored in this study such as its capability to detect extrahepatic arterial supply [25], feeding arteries not identified by CECT [26] or the exclusion of necrotic areas with no contrast uptake for a more precise volumetric assessment of the tumor.

Using the TgVs estimated by each imaging modality and a fictional administered activity of 1 GBq, the corresponding absorbed doses by tumor and non-tumor were calculated for each patient. Consistent with the results obtained for volumetry, ^99m^Tc-MAA-SPECT/CT showed lower differences with the values obtained with ^90^Y PET/CT TgV, than the rest of modalities. Therefore, and as described before [3, 7, 8], the use of ^99m^Tc-MAA-SPECT/CT volumes reduces the risk of underdosing. Even so, using ^90^Y PET/CT as the method to define the actual TgV, the calculated Gy in the tumor were 18% lower than those predicted using ^99m^Tc-MAA-SPECT/CT TgV (median of − 14 Gy). This difference was almost half for absorbed doses by non-tumor liver (median of − 5 Gy). These results are in accordance with other studies founding that ^99m^Tc-MAA-SPECT/CT tends to overestimate posttherapy dosimetry in tumor, being more accurate for the non-tumor liver dosimetric assessment [27, 28]. Regarding the influence that the treatment approach could have in the differences in dosimetry, CBCT showed lower differences for lobar or total treatments than for selective or superselective approaches. As mentioned above, probably the delimitation of TgV in more selective administrations, without clear anatomical boundaries, can be a limitation of this technique. Therefore, more studies are needed to elucidate this.

Another important aspect of the study lies on the added utility that each modality has in RE planning:

- the information obtained from ^99m^Tc-MAA-SPECT/CT was determinant in 71% of the patients due to its capability to define and confirm the TgV in segmental and subsegmental treatments or after flow redistribution; it also helped to detect tumoral areas not receiving ^99m^Tc-MAA with the selected arterial access.

-CBCT was especially useful in 29% of the patients, ensuring the total coverage of the tumor and ruling out the presence of microsatellite lesions that would have changed the selected arterial access. Moreover, thanks to CBCT information it was possible to split the activity according to the volume of tumor perfused by each feeding artery in up to 42% of patients. This approach, which as far as we know has not been published before, enables a better coverage of the microspheres in the target area.

The strengths of this study are worth highlighting. First, all ^99m^Tc-MAA-SPECT/CT TgV were obtained blindly during RE work-up, not knowing the final 90Y-microsphere distribution. Second, the same IR performed RE evaluation and treatment in one day in all patients. This reduces the risk of undesired changes in catheter position and therefore the agreement between the distribution of ^99m^Tc-MAA and the ^90^Y-microspheres is less subject to non-measurable errors.

This study has also some limitations. It is a single-center study involving a relatively small number of patients. Shallow breathing was allowed during SPECT/CT and PET/CT acquisition and breathing motion was not corrected. However, as Bastiaannet et al. [29] described, healthy liver parenchyma suffered only marginally from breathing and collimator effects due to the larger volume, being individual tumors the most affected. Despite these limitations, the results herein presented are promising and can help to plan a more precise and personalized treatment with those imaging methods routinely used during RE work-up.

## Conclusion

^99m^Tc-MAA-SPECT/CT has shown to be a reliable tool to predict the liver volume that will be treated during RE. Its concordance with the TgV obtained with ^90^Y PET/CT has demonstrated to be superior to that obtained with CBCT or CECT, used in current practice. This superior prediction persisted also for segmental and subsegmental infusions performed for a more effective and safer RE. Moreover, the use of ^99m^Tc-MAA-SPECT/CT TgV could have reduced the risk of underdosing with respect to the use of CECT or CBCT TgV. Nonetheless, CBCT provided essential information for a personalized RE planning, ensuring the total coverage of the tumor and, in cases with more than one feeding artery, splitting the activity according to the volume of tumor perfused by each artery. Therefore, the joint use of ^99m^Tc-MAA-SPECT/CT and CBCT optimizes dosimetric planning for RE procedures, enabling a more accurate personalized approach.

## Data Availability

All data collected is anonymized and saved by the first author (Macarena Rodríguez-Fraile). It is available on reasonable request. This study was partially presented in the Annual Congress of the European Association of Nuclear Medicine October 12–16, 2019, Barcelona, Spain. Eur J Nucl Med Mol Imaging 46, 1–952 (2019). https://doi.org/10.1007/s00259-019-04486-2 [30].

## Abbreviations

RE: Radioembolization
SIRT: Selective internal radiation therapy
BSA: Body surface area
mBSA: Modified BSA
TgV: Target volume
TgVs: Target volumes
BS: Bremsstrahlung
REILD: Radioembolization-induced liver disease
IR: Interventional radiologist
NM: Nuclear medicine
CECT: Contrast-enhanced computed tomography
MR: Magnetic resonance
^99m^Tc-MAA: ^99M^Tc-macroaggregated albumin
CBCT: C-arm cone-beam CT
HCC: Hepatocellular carcinoma
CC: Cholangiocarcinoma
MDT: Multidisciplinary team
ROIs: Regions of interest
HPS: Hepatopulmonary shunt
TNR: Tumor/non-tumor ratio
VOI: Volume of interest
VOIs: Volumes of interest
CCC: Lin Concordance Correlation Coefficient
95% CI: 95% Confidence Interval
